# Leveraging artificial intelligence and data science techniques in harmonizing, sharing, accessing and analyzing SARS-COV-2/COVID-19 data in Rwanda (LAISDAR Project): study design and rationale

**DOI:** 10.1186/s12911-022-01965-9

**Published:** 2022-08-12

**Authors:** Aurore Nishimwe, Charles Ruranga, Clarisse Musanabaganwa, Regine Mugeni, Muhammed Semakula, Joseph Nzabanita, Ignace Kabano, Annie Uwimana, Jean N. Utumatwishima, Jean Damascene Kabakambira, Annette Uwineza, Lars Halvorsen, Freija Descamps, Jared Houghtaling, Benjamin Burke, Odile Bahati, Clement Bizimana, Stefan Jansen, Celestin Twizere, Kizito Nkurikiyeyezu, Francine Birungi, Sabin Nsanzimana, Marc Twagirumukiza

**Affiliations:** 1 College of Medicine and Health Sciences, University of Rwanda, Kigali, Rwanda; 2 African Center of Excellence in Data Science, University of Rwanda, Kigali, Rwanda; 3 Rwanda Biomedical Center, Ministry of Health, Kigali, Rwanda; 4 Rwamagana Provincial Hospital, East province, Rwamagana, Rwanda; 5 College of Science and Technology, University of Rwanda, Kigali, Rwanda; 6 The University Teaching Hospital of Kigali (CHUK), Kigali, Rwanda; 7 edenceHealth NV, Kontich, Belgium; 8 Regional Alliance of Sustainable Development, Kigali, Rwanda; 9 Center of Excellence in Biomedical Engineering and eHealth, University of Rwanda, Kigali, Rwanda; 10 Faculty of Medicine and Health Sciences, Ghent University, Ghent, Belgium

**Keywords:** Artificial intelligence, Machine learning, Data science, SARS-COV-2/COVID-19, Rwanda

## Abstract

**Background:**

Since the outbreak of COVID-19 pandemic in Rwanda, a vast amount of SARS-COV-2/COVID-19-related data have been collected including COVID-19 testing and hospital routine care data. Unfortunately, those data are fragmented in silos with different data structures or formats and cannot be used to improve understanding of the disease, monitor its progress, and generate evidence to guide prevention measures. The objective of this project is to leverage the artificial intelligence (AI) and data science techniques in harmonizing datasets to support Rwandan government needs in monitoring and predicting the COVID-19 burden, including the hospital admissions and overall infection rates.

**Methods:**

The project will gather the existing data including hospital electronic health records (EHRs), the COVID-19 testing data and will link with longitudinal data from community surveys. The open-source tools from Observational Health Data Sciences and Informatics (OHDSI) will be used to harmonize hospital EHRs through the Observational Medical Outcomes Partnership (OMOP) Common Data Model (CDM). The project will also leverage other OHDSI tools for data analytics and network integration, as well as R Studio and Python. The network will include up to 15 health facilities in Rwanda, whose EHR data will be harmonized to OMOP CDM.

**Expected results:**

This study will yield a technical infrastructure where the 15 participating hospitals and health centres will have EHR data in OMOP CDM format on a local Mac Mini (“data node”), together with a set of OHDSI open-source tools. A central server, or portal, will contain a data catalogue of participating sites, as well as the OHDSI tools that are used to define and manage distributed studies. The central server will also integrate the information from the national Covid-19 registry, as well as the results of the community surveys. The ultimate project outcome is the dynamic prediction modelling for COVID-19 pandemic in Rwanda.

**Discussion:**

The project is the first on the African continent leveraging AI and implementation of an OMOP CDM based federated data network for data harmonization. Such infrastructure is scalable for other pandemics monitoring, outcomes predictions, and tailored response planning.

## Introduction

In December 2019, a critical respiratory disease from an unknown cause was identified in Wuhan, China [1]. Thereafter, the causative pathogen was discovered as a novel coronavirus and was named the severe acute respiratory syndrome coronavirus 2 (SARS-CoV-2) [1–3]. Since then, the SARS-CoV-2 virus has quickly spread across China and worldwide [1, 4–6]. Since the first released reports of the confirmed cases of the coronavirus disease of 2019 (COVID-19) in Wuhan, China, the whole world has witnessed severe unprecedented mortality and morbidity due to this disease resulting in serious public health emergencies that defined the disease as a global pandemic [7–9]. Although the initial outbreak of the pandemic in Africa was delayed due to less rapid importation, the risks posed by a significant outbreak remained high due to Socio-economic and health service capacity issues [10–12]. The first patient diagnosed with COVID-19 was detected in Rwanda in March 2020 [13]. After detection of the first case, different public health measures have been implemented by the Rwandan government from total lockdowns, inter-district lockdowns, localized lockdowns and others [14, 15]. The classic public health measures to prevent COVID-19 were also emphasized such as the mandatory wearing of masks, social distancing and regular handwashing [16, 17]. Rwanda has also joined the rest of the world to secure vaccines against COVID-19 [18]. Despite all efforts, Rwanda has exceeded 80,000 cases of COVID-19 with more than 1000 deaths and numbers keep increasing [13].

In addition, with the apparent unpredictability of the increase or decrease of COVID-19 cases, both the decision-makers and the general population live in an uncertain situation [19]. Accurate short-term forecasting of COVID-19 spread plays an essential role in improving the management of the overcrowding problem in hospitals and enables appropriate optimization of the available resources [20]. This forecasting effort helps also to reduce the burden of COVID-19 in terms of planning and adjustment of public health measures. This study, the first to our best knowledge in Rwanda, has proposed the building of data hubs which will later be used to design COVID-19 prediction models using Artificial intelligence (AI) and Machine Learning (ML) techniques. Recently deep learning methods have gained particular attention in time-series modeling and analysis because of their outstanding generalization capability and superior nonlinear approximation [20–23].

In Rwanda, the use of AI and data science techniques is motivated by a large implementation of electronic medical record (EMR) systems in the health care facilities which makes data accessible. However, the data is fragmented, as health facilities are using different EMRs, and the COVID-19 data were not systematically collected. To effectively visualize and re-use the data, there’s a need to create a centralized common data model using already collected data for both COVID-19 and other medical conditions. In addition to understanding the spread of COVID-19 data, prospective understanding of respect of public health measures is also mandatory. For example, in the study by Barak et al., they emphasized that obtaining information on symptoms dynamics is of great importance to control the complications of the disease in the population [24]. In the current study, we will assess longitudinally COVID-19 related symptoms and preventive measures taken in each area. This project will leverage Artificial Intelligence (AI), Machine Learning (ML) and other Data Science (DS) techniques to create a scalable framework for inventorying, harmonizing and federating the accumulated data from COVID-19 patients and converting it to a standardized data format so that it can be used as part of wider studies on the disease. The harmonized data will consist of COVID-19 diagnosed/serotyped patient data and non-infected individuals from electronic health records (EHRs) of different hospitals and databases of testing centers (positive and negative results). In the second phase of the project, we will collect new data longitudinally including patient-reported outcome (PROs). Those newly collected longitudinal data will be in a similar standardized model by design and could be potentially be linked to federated data.

The project outcome is to leverage all federated data to drive evidence which will fulfil Rwanda's needs and priorities in predicting and monitoring the burden of COVID-19 pandemic in the Rwandan community, on hospital admissions related to COVID-19 and overall COVID-19 infection rates. The generated evidence will also monitor the impact of different public health measures on the COVID-19 pandemic evolution in Rwanda.

## Materials and methods

### Study setting

The study sites will include 13 hospitals (The University teaching hospital of Kigali, Butare University teaching hospital, Ngoma regional hospital, Ruhengeri provincial hospital, Muhima district hospital, Kibagabaga district hospital, Nyamata district hospital, Nyagatare district hospital, Kinihira district hospital, Kigeme district hospital, Kirehe district hospital, Gisenyi district hospital and Gihundwe district hospital); two health centers (Remera health center and Nyamata health center); and one centralized dataset gathering 9 COVID-19 test centers (Kanyinya, Rwankeri, Gatenga, Kicukiro, ASPEK-Ngoma, Kigali Transit Centre, Rugerero, Kabgayi and Rusizi). These study sites have been selected to represent all four provinces of the country and the Kigali city for prediction and generalizability purposes.

### Inclusion and exclusion criteria

The included health facilities were selected based on the availability of EMRs at hospitals. For the community surveys, all participants aged 18 years and above will be eligible to enter the study. Participants will only be excluded if they have no eligible household phone contacts. According to the national regulations, all participants will provide consent to participate in the study. The consent will be electronically signed and embedded into a mobile application used for surveys.

### Study design

**Fig. 1:**
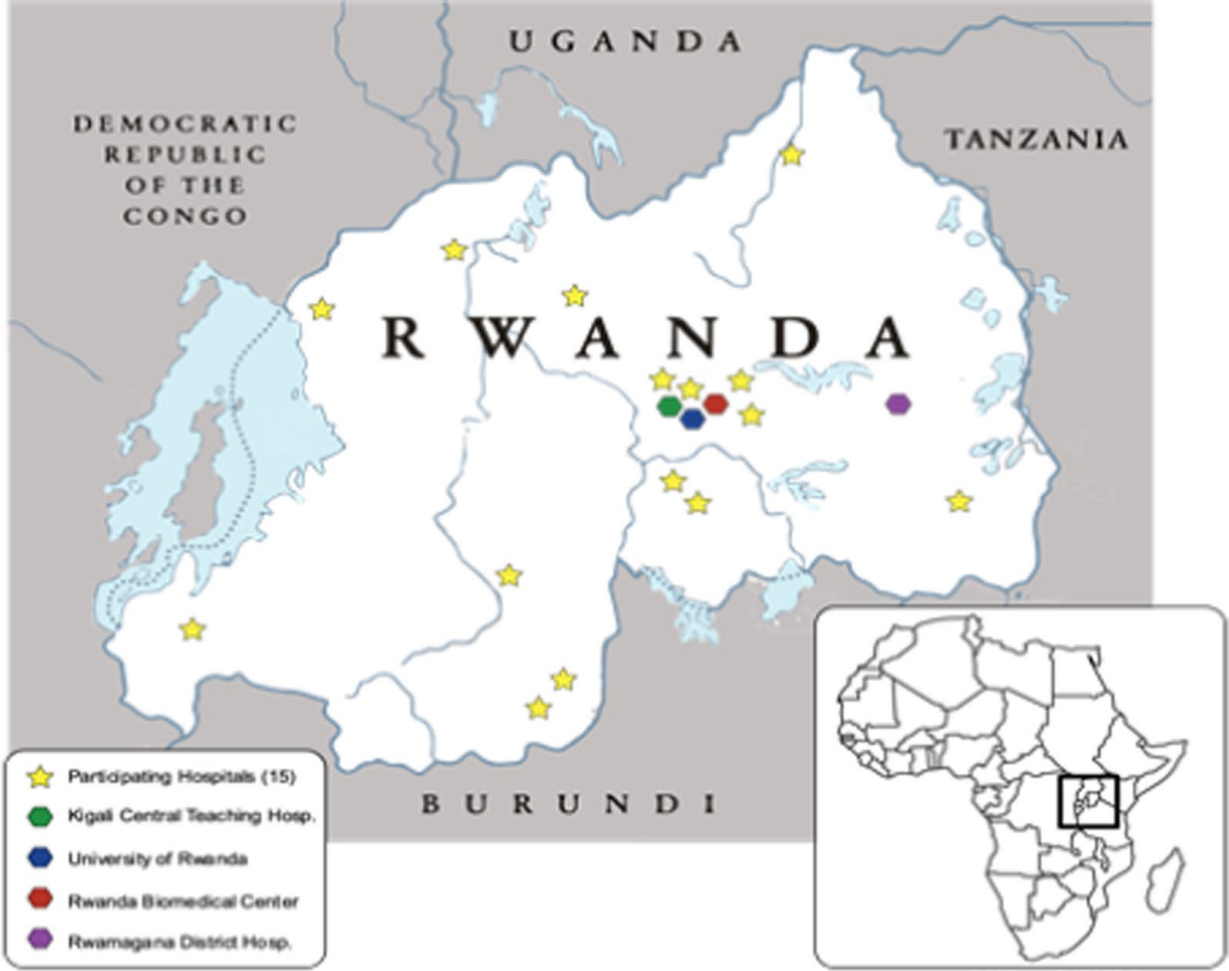
LAISDAR participating institutions

The LAISDAR (Leveraging Artificial Intelligence and Data Science Techniques in Harmonizing, Sharing, Accessing and Analyzing SARS-COV-2/COVID-19 Data in Rwanda) project is a federated data network, based on the Observational Medical Outcomes Partnership (OMOP) Common Data Model (CDM), as well as on open-source Observational Health Data Sciences and Informatics (OHDSI) tools for data analytics and network integration, and R Studio and Python. As demonstrated in Fig. 1, the network will include several hospitals, whose EHR (Electronic Health Records) data will be harmonized to OMOP CDM, and enriched both with COVID-19 test results, COVID-19 survey results from a national database, and the results of the community surveys. An initial proof-of-concept (POC) implementation was set up and tested, which included the central LAISDAR instance and 2 data nodes—one on a Mac Mini and one on an AWS EC2 (Amazon Elastic Compute Cloud) instance.

There are 2 different open source EHR systems used by the participating hospitals; OpenMRS [25] and OpenClinic GA [26]. Therefore, two different ETL (Extract Transform Load) processes will be implemented in order to have as few local adaptations per hospital as possible.

Enrichment of EHR data is part of the ETL process, whereby available COVID-19 test and survey results will be retrieved from a central repository over a secure interface. One critical challenge with this step is consolidating individual patients; different person identifiers (national ID, mobile number, name, address), or combinations thereof, are used across different systems. We envision generating unique identifiers based on the available keys to facilitate a reliable and reproducible matching of records from the different source systems.

The integration of the sites with a central hub will be accomplished by using the open-source version of Arachne, which provides a platform for performing network studies, integrating OHDSI standards and tools.

Software deployments at the participating hospitals will rely on Docker-based containerization; this approach ensures consistent and reproducible installation across the different sites. For most participating hospitals, a pre-configured Mac Mini will be provided with the complete LAISDAR Dockerized software suite.

### Conceptual framework for hospital EHRs harmonization

**Fig. 2:**
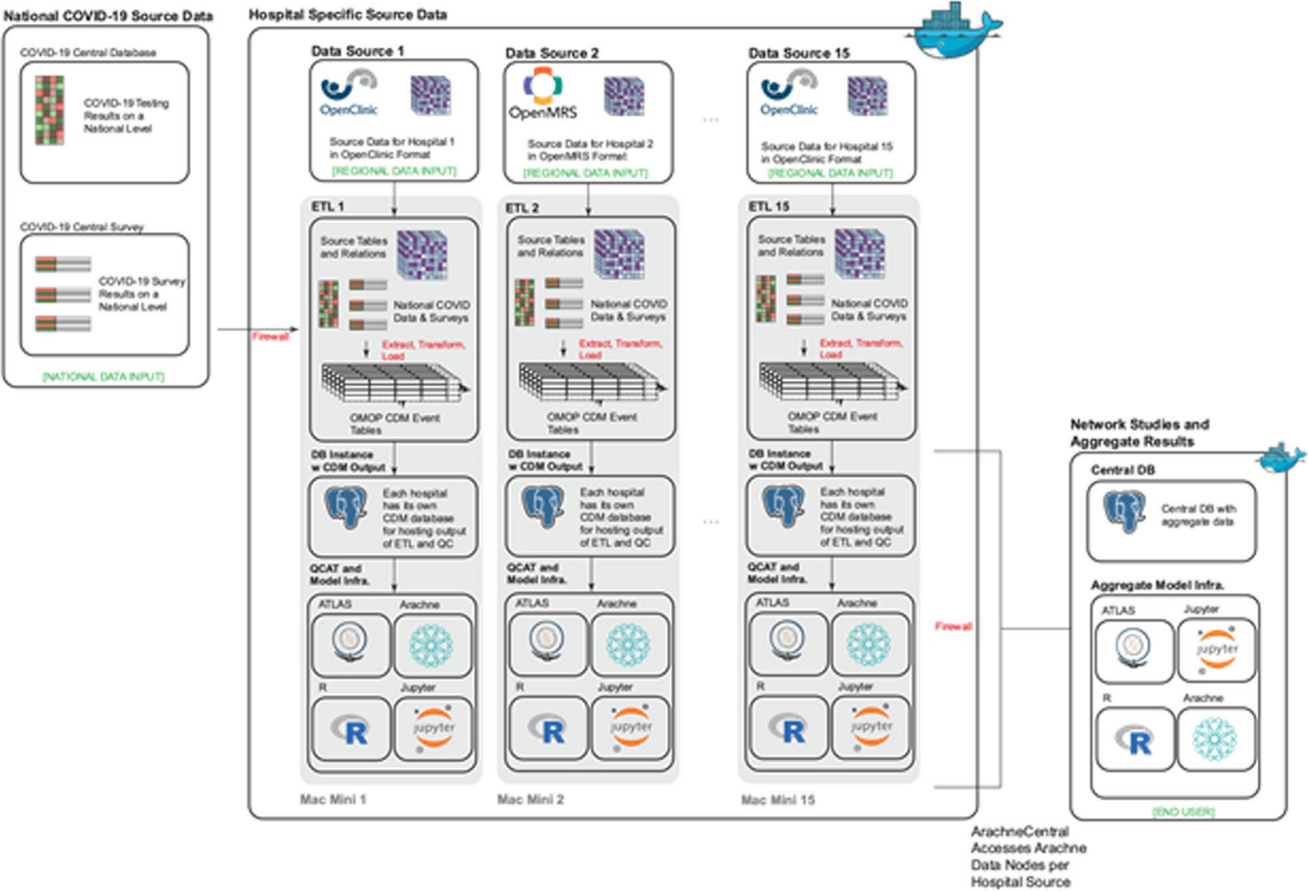
Overall LAISDAR architecture for data harmonization

The conceptual framework is presented in Fig. 2. After inventorying existing datasets this project will set up an infrastructure for data harmonization where novel techniques will be developed including the data access and data analysis interface where we will mix the existing methods and innovative techniques. Within this conceptual framework it’s planned to include: a data catalogue describing the different data sources, the Arachne central hub, a central OHDSI Atlas instance, a central database, as well as R Studio and Jupyter.

### Data collection, analysis and management

The study will involve four main steps with regards to data collection, analysis and management:

#### Step 1: Data gathering/collection

This work will start by inventorying and gathering various scattered data in Rwanda including existing data from the first 24 months of the COVID- 19 pandemic in Rwanda (the first case was identified in march 2020) together with other hospital data completed with new community survey data.

It’s anticipated to have different formats of data sources ranging from Covid-19 related data registered in Excel documents, via data sources containing Minimum Clinical Data (MCD) in DHIS2 [25] and other systems, to more granular Electronic Medical Record (EMR) data in Open Clinic GA [26], OpenMRS [25] and other EMR systems. We will start by mapping full hospital patients’ records, focusing on 15 health facilities located in regions with a high number of COVID-19 patients and completing with other isolated datasets.

The new data collection (community surveys) will use standardized protocol and questionnaires and will be done according to a longitudinal approach. The participants will be randomly sampled following the sampling frame used for the recent Rwanda Demographic Health Survey (based on the fourth Rwanda Population and Housing Census RPHC) provided by the National Institute of Statistics of Rwanda (NISR) [27]. This sampling frame is a complete list of districts covering the whole country. The data collection is done through bi-weekly phone call, 6 phases planned (starting by December 2021), involving 30 well trained data collectors, one per district supervised by 10 investigators. The minimum sample size required was estimated at 107 people per each of 30 administrative districts in Rwanda. To anticipate on consent refusal and drop outs, we doubled this number making 214 participants in each district. If the participant has a medical file in participating hospitals, or in other COVID-19 testing dataset, the datasets will be linked with possibilities of other linkage data request in future.

The sample will proportionally include males and females based on the number of inhabitants. Each participant will receive a mobile fee connection and internet bundle each week to allow data collection. To mitigate the expected gap of the gender digital divide but also of selected persons without a mobile phone anymore, the consortium established mitigation measures, including but not limited to, leveraging the community healthcare workers (CHWs). Each village in Rwanda has a CHW who participates in various ministry of health (MoH) programs and they have all received the mobile phones from MoH. If we select a respondent without a mobile phone we will liaise with the nearest CHW to reach out to him.

The questionnaires (which will be translated into 3 languages, Kinyarwanda, English and French) include 10 modules (at least 8 of them has to be fulfilled by the project): (1) Demographics; (2) Face mask use; (3) Hand hygiene; (4) Respect of social distancing measures and risk minimization measures; (5) Recent risk situations exposures and COVID-19 measures. On the outcome side, the collected data will include; (6) Coronavirus like-Signs and symptoms; (7) Mental health indicators (based on General anxiety disorder-GAD); (8) Social-economic impact (based on loss of income, or categories) and 9) Covid-19 test results [28].

### Gender considerations

Gender facets are found in all COVID-19 consequences including morbidity in general, gender based violence and mental health problems in particular and Socio-economic situations related to lock-down and other COVID-19 consequences. Social and cultural factors related to gender such as specific considerations for some collected data elements will be addressed as well, eg. reproductive health data, the usage of gender-sensitive research questions and gender-impartial language. Moreover, the sampling will pay special emphasis to gender proportional balance while collecting new COVID-19 data and gender key output/aspects will be driven from data analysis.

#### Step 2: Infrastructure for data harmonizing (developing novel techniques)

For data harmonization, the custom-designed ETL scripts will be developed per data source to extract, transform and load the source data to an OMOP CDM database instance. In the early stages, when the hospital EHRs are not yet harmonized, we will also use synthetic data approaches to help automate harmonization processes. The data owner-side infrastructure will include the OMOP CDM database instance, the Arachne client, the OHDSI Atlas [29] analytical tool, R Studio [29], and Jupyter [29]. The data harmonization process converts the observational data from the format of the source data system to the OMOP CDM supported by the OHDSI organization.

#### Step 3: Infrastructure for data access, query, and data analysis (mixing existing methods and innovative techniques)

The central platform data access, query, and data analysis, will handle the participating data sources. The central site will use Arachne for the central portal with the data and study catalogues, but also a PostgreSQL database, an OHDSI Atlas analytical platform instance and an R Studio instance. Additional tools can be added like a Jupyter server instance. As a standard, the database will include an OMOP CDM schema, and additional schema(s) to support the central data catalogue. At the beginning of the harmonization process, as the data from hospitals EHRs will be not yet available, this project will use synthetic data to help automate harmonization processes and training models, specially we will use the OHDSI community’s available mock-up data (like Synthea) to train different algorithms /models before we use them on real data.

The Arachne central server setup will allow central management of network studies, with tight integration with the OHDSI tools such as Atlas. The Arachne data catalogue will incorporate the Achilles output from each participating site; the Achilles tool generates a profile of the participating sites’ data on an aggregate level, which will allow a central view of the descriptive statistics for each site. The R Studio and Jupyter instances will allow the development and testing of R scripts as part of a study design, or to analyze data collected from data source sites as part of studies.

#### Step 4: Data analysis and interpretation (mixing existing methods and innovative techniques)

Within this work, further data analysis is foreseen. It’s known that the federated datasets are challenging to analyze with traditional statistical methods, because they are, like other real-world-data (RWD), 1) collected without any intention for being used in research; 2) incomplete and not cleaned and 3) collected in a sporadic way, not pure longitudinal approach so no way to derive cohort-like data from them. On such data the use of artificial Intelligence and Machine Learning (AI/ML) in analysis has been advised. However, within this work no single model of AI/ML has selected upfront as the data gathered was unlikely similar to others found in the literature and this was a pioneer work in Rwanda. Therefore, we planned first to evaluate the performances of different deep learning methods before deciding which model fits to the gathered data. The use of AI/ML for data analysis will be fully described in further work as well as the contribution of previous work on a Capsule Network based framework [30].

In prediction models, the sequential reproduction number R(t) will be estimated using the Bayesian approach on the Extended SEIR compartmental model. The Bayes rule is used to update the beliefs about the true R(t) based on model predictions and new cases that have been reported each day.

#### Model definition

**Fig. 3:**
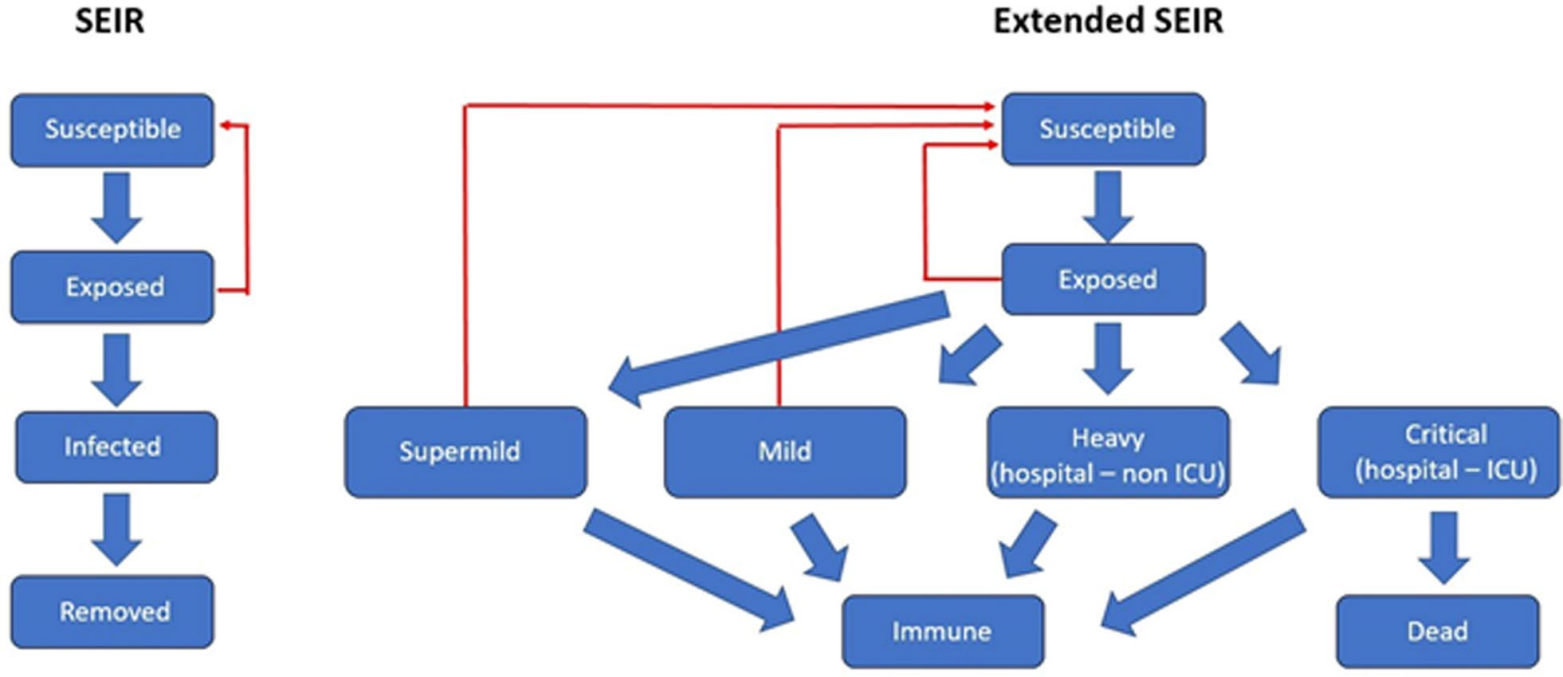
Prediction model definition [32]

We will use an extension of the SEIR model (Fig. 3) inspired by the previous works [31]. This model splits the population into different categories, i.e. susceptible, exposed, infected and removed. The latter two categories are further broken down into super mild, mild, heavy and critical for the infected part of the population, whereas the removed population indicates the immune and dead fraction. A super mild infection refers to the category of asymptotic people who are infected but are unaware of their own infection. Recent figures from Chinese scientists put this number at 86% of all infections [31].

Transitioning between different fractions of the population is indicated by the arrows and its rates are expressed by parameters in the model. The two most important parameters in such a model are: (1) the incubation period and (2) rate of virus spread. Other parameters include the odds of having a super mild, mild, heavy or critical infection. For each type of infection, there is an infectious period, etc. All parameters except one were gathered from the available literature on coronavirus. The parameter that remained to be calibrated is ‘beta’, which determines the rate of transitioning individuals from susceptible to exposed. Beta can be interpreted as the degree of social interaction or the amount of exposure to the virus. It is this parameter that is targeted when governments impose restrictions on their citizens. We will, therefore, focus on this parameter. Finally, a documented mathematical model will be discussed at a later stage, at the beginning of the project implementation.

### Study limitations

We acknowledge the limitations imposed by a federating network approach used by LAISDAR project, where the data remains at each participating hospital’s site, rather than the patient-level data being pooled in a central location. There are different approaches to accommodate a federated approach for the ML methods described here; the main challenge relates to training any model across all the considered remote data, as it must be done in a federated manner.

Also, the project being pioneer work in this field in Rwanda, we expect some other limitations: data incompleteness, missing data and/or data inaccuracy. Additionally, some data are still captured at health facilities as free text and will not be exploitable by our work, as no Natural Language Processing (NLP) technique will be applied. Finally, this work will work only with Health Facilities where Electronic Health Records is used, leaving behind other health facilities still using paper-based records. This might have an impact on generalizability of the study findings. However, the large sample size expected, the robust sampling methods and federating approach will mitigate this potential bias.

### Sustainability and scalability of the project

This project will set up infrastructure both non-material (federation network of hospital data, harmonized data and OMOP data layer in each hospital) but also materials (equipment: servers, workstations). Those infrastructures will stay in place and can be re-used for future projects. The maintenance of this infrastructure is in the Ministry of Health (MoH) of Rwanda hands, through the key partner on this project the Rwanda Biomedical Center, an implementation body of MoH.

The existing infrastructure can be also scalable either to focus on other infectious diseases (Malaria, Tuberculosis) or non-communicable diseases (Hypertension, metabolic diseases) or other emerging pandemics in future. However, the scalability might be challenged by lack of local funding needed for staff/researchers training, new equipment to be purchased etc.

### Study expected results, outcomes and impact

#### Technical infrastructures

An initial proof-of-concept (POC) implementation was set up and tested early in the project, which included the central LAISDAR instance and 2 data nodes—one on a Mac Mini and one on an AWS EC2 instance. The data nodes were set up using Docker containers providing the following services: a (PostgreSQL) OMOP CDM database, Atlas/web API, Achilles and Arachne (connected to the Arachne Central instance). The central server was set up with Arachne Central, where the Data Catalogue was configured, and studies were created and executed for testing the integration at the data nodes. The objective of the POC was to test the integration layer (Arachne), as well as to demonstrate the overall process flow for network studies; these objectives were met.

The next phase of the development is well underway, which includes the completion of the ETL implementations, and the integration with the central COVID-19 test and survey results.

The first phase of the project will include 15 health facilities with plans to include additional hospitals in a later phase of the project.

#### Capacity building through training.

This project will mainly contribute to research and capacity building through training staff before and during the project both at UR and at participating hospitals. The planned training includes:1) data mapping infrastructure; 2) training on surveys instruments and 3) training on sensitive patient data handling, data harmonization, interoperability and medical terminology: A team from Ghent University (Belgium) has trained the Rwandan research team on OHDSI OMOP CDM mappings including terminology and coding.

#### Clinical, epidemiological, mental and socio-economic outcomes results:

This project will yield prediction models for the burden of COVID-19 in the community but also the potential impact on hospital admissions or overall infection rates and the impact of various public health measures on (1) the pandemic evolution in the country; (2) on the social-economic situation, (3) and on the mental health (stratified by gender and other vulnerable groups). As intermediate results, the community survey will be analyzed separately on all scopes including descriptive statistics of Socio-economic impact, epidemiology, mental and clinical outcomes. For Socio-economic outcome, the variables to be analyzed are related to the effect of covid-19 on livelihood with a focus on its effect on basic needs (food, medical, care, school fees and transport), income, employment and saving. A logistic model will be formulated and used to analyze the Socio-economic characteristics of people who have been experiencing some economic difficulties due to the COVID-19 situation.

On epidemiological aspects we will investigate the prognosis factors associated with clinical outcome of COVID-19 burden in Rwanda, and the drivers of COVID burden in Rwanda.

Regarding the gender and mental health multiple axes of research are planned including (1) the longitudinal study on stigmatization and associated factors during the COVID-19 pandemic in Rwanda; (2) Behavioral/ Gender based violence outcome of COVID-19 in Rwanda; (3) longitudinal study on mental health wellbeing and associated factors during the COVID-19; and others.

Finally, a cultural analysis is planned to investigate how Rwandans deal with the COVID-19 pandemic and the related control measures.

## Conclusion

To the best of authors’ knowledge, this project is the first on the African continent to implement data harmonization on COVID-19. The design and implementation of an OMOP CDM based federated data network for COVID-19 related studies in Rwanda will provide researchers in Rwanda and elsewhere with the tools and data access needed to better track the disease, predict outcomes, and plan appropriate responses.

The chosen architecture lends itself to expansion to additional hospitals or other data sources, should there be a need. Building the LAISDAR infrastructure on the open-source OMOP CDM data model and utilizing OHDSI tools and other open-source tools facilitates easy involvement of new partners. In addition, these choices provide opportunities for participation in other OHDSI based network studies around the world.

## Data Availability

This article is a research proposal. The dataset generated for some of the phases of the study have not yet been analysed but will be made available from the corresponding author on reasonable request.

## Abbreviations

AI: Artificial intelligence
LAISDAR: Leveraging artificial intelligence and data science techniques in harmonizing, sharing, accessing and analyzing SARS-COV-2/COVID-19
CHUK: The University Teaching Hospital of Kigali
SARS-COV-2: Severe acute respiratory syndrome coronavirus 2
COVID-19: Coronavirus disease of 2019
OHDSI: Observational Health Data Sciences and Informatics
OMOP: Observational medical outcomes partnership
CDM: Common data model
EHR: Electronic Health Records
EMR: : Electronic Medical Records
ETL: : Extract transform load
DS: Data science
ML: : Machine learning
PROs: With patient reported outcome
CHWs: Community health workers
MoH: Ministry of Health
NISR: National Institute of Statistics, Rwanda
R&I: Research and Innovation
RWD: Real World Data
UR: University of Rwanda
SEIR: Susceptible-Exposed-Infected-Removed
API: Application Programming Interface
GAD: General anxiety disorder
POC: Proof of concept
MCD: Minimum Clinical Data
WHO: World Health Organization
